# Extended Anterolateral Thigh Flaps for Reconstruction of Extensive Defects of the Foot and Ankle

**DOI:** 10.1371/journal.pone.0083696

**Published:** 2013-12-23

**Authors:** Lifeng Liu, Xuexin Cao, Lin Zou, Zongyu Li, Xuecheng Cao, Jinfang Cai

**Affiliations:** Orthopeadic Department, the General Hospital of Jinan Military Command, Jinan, China; Sapienza, University of Rome, School of Medicine and Psycology, Italy

## Abstract

The anterolateral thigh flap has been used for coverage of defects in the foot and ankle for years. Conventional extended anterolateral thigh flaps do not undergo thinning procedures, which limit their application. Here, a clinical series of 24 patients is reported in which extended anterolateral thigh flaps were used for posttraumatic foot and ankle reconstruction. Of the 24 flaps, 14 were simple extended anterolateral thigh fasciocutaneous flaps and 10 were thinned extended anterolateral thigh flaps. One artery and two veins, including a superficial vein and an accompanying vein, were anastomosed to vascularize each flap. Follow-up of the 24 patients ranged from 10 months to 4 years postoperatively. All 24 flaps survived successfully, except one case that had partial flap necrosis distally due to excessive thinning. The cutaneous flap territory ranged from 250 cm^2^ to 400 cm^2^ (mean, 297 cm^2^). Only one patient received a debulking procedure. No ulceration occurred in any of the flaps due to contact with the shoe. The extended anterolateral thigh flap is a good alternative for extensive soft tissue defects of the foot and ankle. This study also supports the high reliability and excellent vascular supply of moderate thinned extended ALT flaps.

## Introduction

Since the conquest of the upright position, the foot has gained more importance as an organ that allows humans to stand up, walk, run, jump and climb. Without the overlying skin and soft tissue on the foot and ankle, they could not support the weight of the body. So the importance of the functional reconstruction of the foot and ankle was highlighted [1]. Reconstruction of extensive defects in the foot and ankle, especially those involving exposure of bone, joint or tendon, is a major surgical challenge. There are several reconstructive options open to the clinician, including local cutaneous flaps, pedicled fasciocutaneous flaps and microsurgical flaps [2]–[4]. Local cutaneous flaps are usually not applicable because of the limited amount of soft tissue that can be transferred. Because the proximal ipsilateral leg has a large skin area that may provide a tissue source, sural flaps have been used for reconstruction of foot and ankle in recent years [5]. However, the sural flap has its own disadvantages, including restricted rotation reach, flap congestion, flap thickness and partial flap failure [6], [7]. Moreover, sural flap elevation depends on good posterior calf tissue, which is not always available in cases with severely traumatized feet and ankles.

For extensive defects with bone or tendon exposure in the lower extremity, reconstruction with free flaps is often an alternative to salvage the foot. The anterolateral thigh (ALT) flap, as first described by Song et al [8], is a versatile and reliable flap based on the cutaneous perforators from the lateral femoral circumflex artery. This flap has gained popularity in recent decades and has already become a routine technique for foot and ankle reconstruction [9], [10]. Extended anterolateral thigh flaps are usually defined as having a vascular territory ≥ 240 cm^2^. Many studies have proven that the anterolateral thigh flap can be safely extended to include adjacent vascular territories perfused by a single perforator from the lateral femoral circumflex artery [11], [12]. Its moderate thickness and large cutaneous vascular territory allows aesthetic and functional reconstruction of extensive defects of the foot and ankle. In this study, the authors present their experience with this technique.

## Patients and Methods

All cases were obtained from the General Hospital of Jinan Military Command.The research followed the ethical guidelines of the directive 2009/18 of the Ministry of Health, P.R.China. Protocols applied in this study and the publishment of patients' details have been approved by the Hospital Ethical Committee of the General Hospital of Jinan Military Command(90MH-HEC). And written informed consent for publication of their medical details was obtained from patients and their relatives. The method reported here is standard practice in our local hospital. We attending doctors analyze the state of defects and formulate strict preoperative communication for each patient to make the clinical decisions and administered the intervention by ourselves.

### Patients

From May 2003 to June 2011, we treated 24 patients for a variety of extensive traumatic soft-tissue defects of the foot and ankle with extended anterolateral thigh flaps. Of the 24 patients, 17 were men and 7 were women, with ages ranging from 17 to 59 years (average age: 38 years). All injuries were caused by trauma. The defect areas were all located in foot and ankle and ranged from 19×13 cm to 25×16 cm. Patient details are shown in Table 1.

**Table 1:** Statistical description of case series (N = 24 procedures in 24 patients).

Pt. No	Age/sex	Defect Location	Type of flap	Flap size* (cm)	Area(cm^2^)	No. of perforators	Complication	Followup(months)
1	42/Male	Hind foot and heel	Extended ALT musculocutaneous perforator flap	20×15	300	1	None	15
2	32/Male	Dorsum and sole	Extended ALT septocutaneous perforator flap	20×16	320	1	Flap thickness	36
3	41/Female	Dorsum	Extended ALT musculocutaneous perforator flap	18×14	252	1	None	21
4	35/Female	Ankle and dorsum	Thinned extended ALT musculocutaneous perforator flap	25×13	325	2	None	14
5	51/Male	Ankle and dorsum	Extended ALT septocutaneous perforator flap	22×12	264	1	Wound infection	32
6	44/Female	Ankle	Thinned extended ALT musculocutaneous perforator flap	19×15	285	1	None	42
7	31/Male	Dorsum and sole	Extended ALT musculocutaneous perforator flap	20×14	280	1	None	25
8	27/Male	Dorsum	Thinned extended ALT musculocutaneous perforator flap	18×16	288	1	None	15
9	17/Female	Dorsum	Extended ALT septocutaneous perforator flap	18×14	252	1	None	10
10	51/Male	Ankle	Extended ALT septocutaneous perforator flap	20×14	280	1	None	48
11	47/Male	Dorsum	Thinned extended ALT musculocutaneous perforator flap	22×15	330	1	Partial flap necrosis	24
12	36/Male	Dorsum	Extended ALT musculocutaneous perforator flap	18×15	270	2	None	19
13	42/Male	Dorsum and sole	Thinned extended ALT musculocutaneous perforator flap	25×16	400	3	None	20
14	29/Female	Degloving forefoot	Extended ALT musculocutaneous perforator flap	20×15	300	1	None	24
15	46/Male	Dorsum and sole	Extended ALT septocutaneous perforator flap	20×15	300	2	None	10
16	21/Male	Dorsum	Extended ALT musculocutaneous perforator lap	19×13	247	1	None	15
17	51/Male	Ankle and dorsum	Thinned extended ALT musculocutaneous perforator flap	25×14	350	2	None	19
18	26/Male	Hind foot	Thinned extended ALT musculocutaneous perforator flap	18×15	270	1	None	14
19	32/Male	Hind foot and heel	Extended ALT musculocutaneous perforator flap	20×15	300	2	Skin grafting loss	36
20	31/Male	Hind foot and medial ankle.	Extended ALT musculocutaneous perforator flap	24×16	388	1	Fatigue of climbing	30
21	48/Female	Dorsum	Thinned extended ALT septocutaneous perforator flap	19×15	285	2	None	10
22	59/Male	Degloving forefoot	Thinned extended ALT musculocutaneous perforator flap	22×15	330	2	Wound infection	15
23	41/Male	Ankle	Extended ALT septocutaneous perforator flap	20×13	260	1	None	18
24	33/Female	Dorsum	Thinned extended ALT musculocutaneous perforator flap	18×14	250	2	None	20

* Length by width.

### Operative Technique

The dimensions of each flap depended on the size of the defect. The skin paddle was marked on the anterolateral face of the thigh along a line connecting the anterior superior iliac crest to the superolateral border of the patella, according to a template made from the defect. Preoperatively, a handheld Doppler probe was used to locate the position of the perforators, which were usually around the midpoint of the line. We aimed to center the skin paddle of the flap over the selected perforator to maximize perfusion to the most distal portion of the flap.

The flap was harvested with the patient in a supine position. Flap dissection began with an incision along the medial border of the flap down to the fascia, which then was incised and dissected over the rectus femoris muscle. The subfascial dissection was continued laterally to identify the septocutaneous or the musculocutaneous perforators of the descending or transverse lateral circumflex femoral vessels. Septocutaneous perforators run in the intermuscular space between the rectus femoris and vastus lateralis muscles. Musculocutaneous perforators, which penetrated the vastus lateralis muscle, were followed by intramuscular dissection and a small cuff of the muscle was preserved to protect the perforator. The remainder of the flap was then incised and the vascular pedicle was cut distally from the point where the nutrient vessel of the rectus femoris muscle branched. If the ALT flap was thinned before transplantation, a small deep fascial cuff was left around the perforators to limit the risk of damage to the vessel. The flap was checked for viability and then transferred to the defect with vessel anastomosis. One artery and two veins, including a superficial vein and an accompanying vein, were anastomosed to vascularize each flap.

All donor sites were closed by split-thickness skin grafting. Drainage was placed under the flap. The extremity was covered with a soft dressing postoperatively, leaving the flap exposed to monitor perfusion. Two days later, the dressing was changed and the drainage was removed. All patients were non-weight bearing postoperatively until their associated fractures were fully healed.

### Outcomes Assessment

Outcomes assessed included flap complications, wound complications, donor site complications, contour adaptation and need for additional procedures.

## Results

Follow-up of the 24 patients ranged from 10 months to 4 years (average: 22 months) postoperatively. Of the 24 flaps, 14 were simple extended anterolateral thigh fasciocutaneous flaps and 10 were thinned extended anterolateral thigh flaps. Septocutaneous perforators were found in 7 of 24 cases (29.2%). Musculocutaneous perforators were found in 17 cases. The dimensions of the flaps ranged from 19×13 cm to 25×16 cm (247 cm^2^ to 400 cm^2^, mean 297 cm^2^). All flaps survived successfully. One case had partial flap necrosis distally due to excessive thinning, which was healed with skin grafting(case number 11 in Table 1). Two patients acquired wound infections that healed with dressing changes and administration of intravenous antibiotics. Skin graft loss at the donor site occurred in one patient and was managed by a second skin grafting. One of the early patients underwent a debulking procedure for a degloving whole flap, which was then transitioned into a full thickness skin grafting. No ulceration occurred in any of the flaps due to undesirable contact with the shoe. At an average of 2.7 months, patients achieved full weight bearing and ambulation. Only one patient complained of fatigue while ascending and descending stairs.

## Case Report

### Case 1

**Figure 1:**
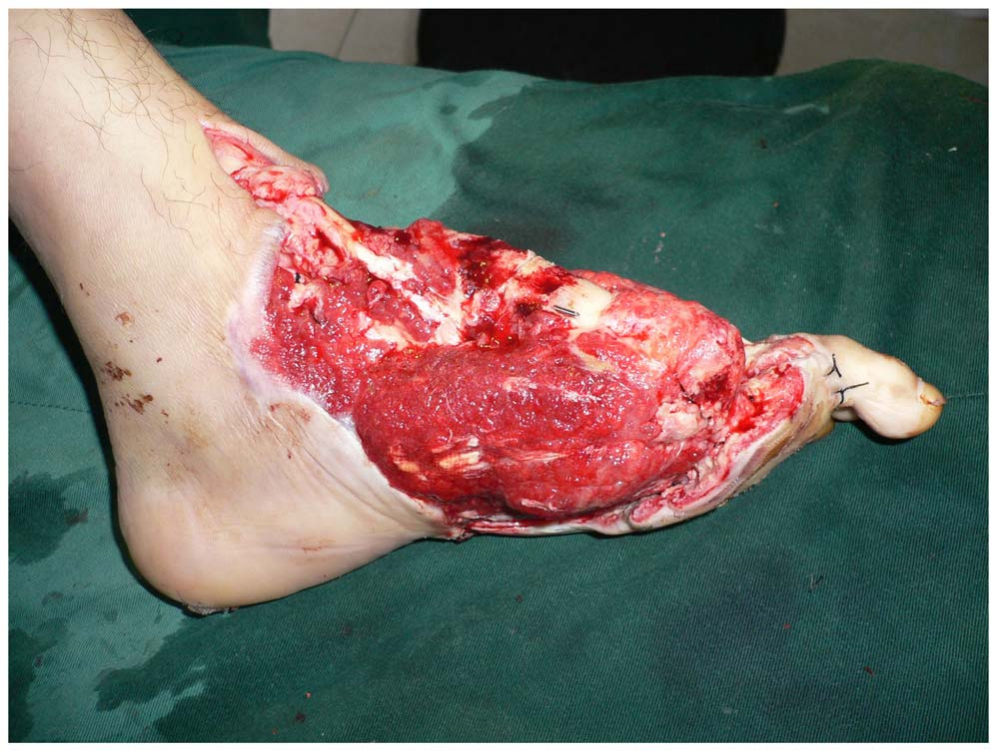
Preoperative view of extensive soft tissue defect of the dorsum and weight-bearing sole of the foot with exposure of the transverse tarsal joint.

**Figure 2:**
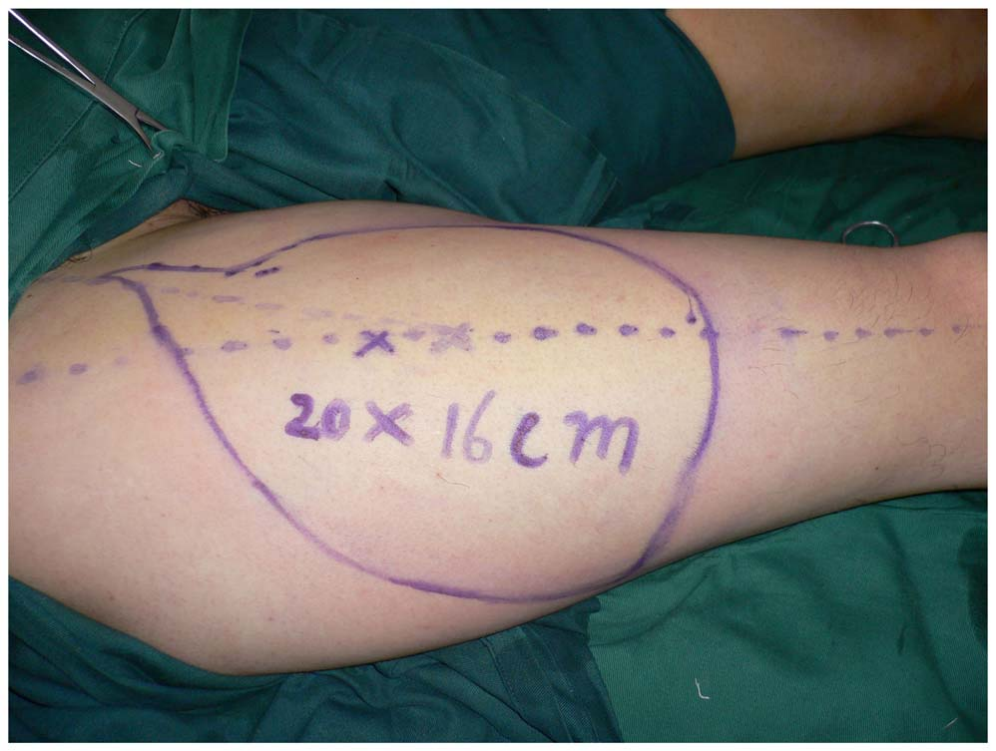
The ALT flap (20×16 cm) was designed according to a template made from the area of the defect.

**Figure 3:**
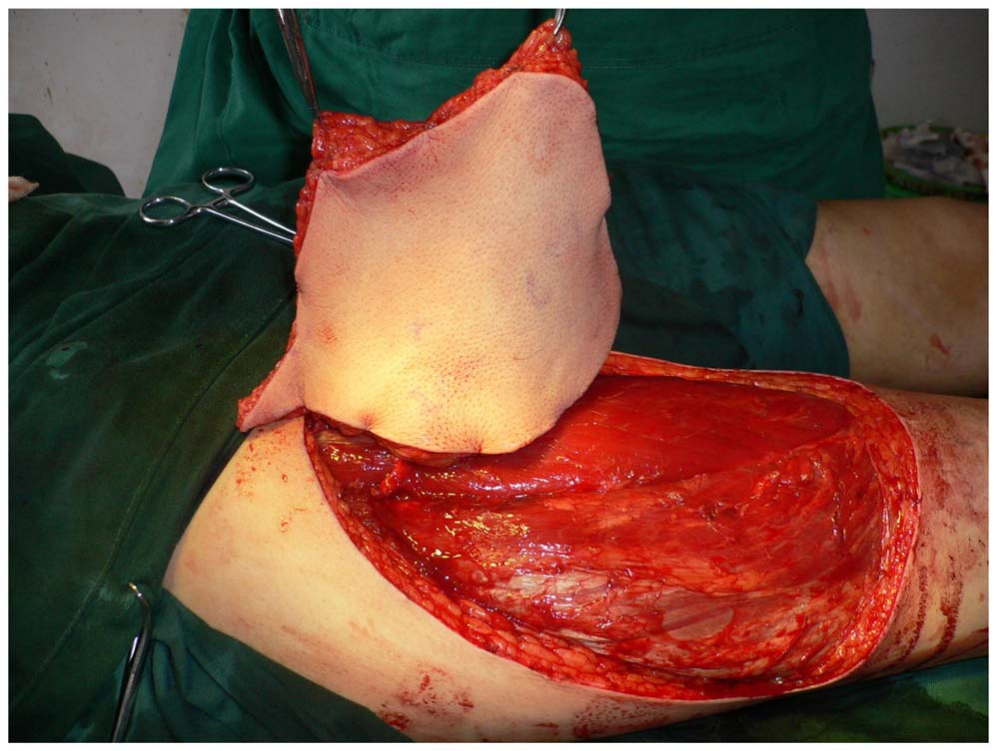
Intraoperative view after elevation of the ALT septocutaneous perforator flap.

**Figure 4:**
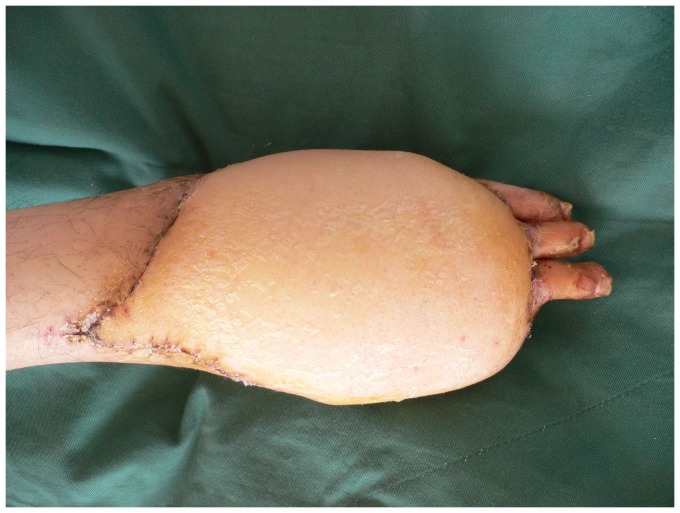
Appearance 15 days after operation with complete flap survival.

**Figure 5:**
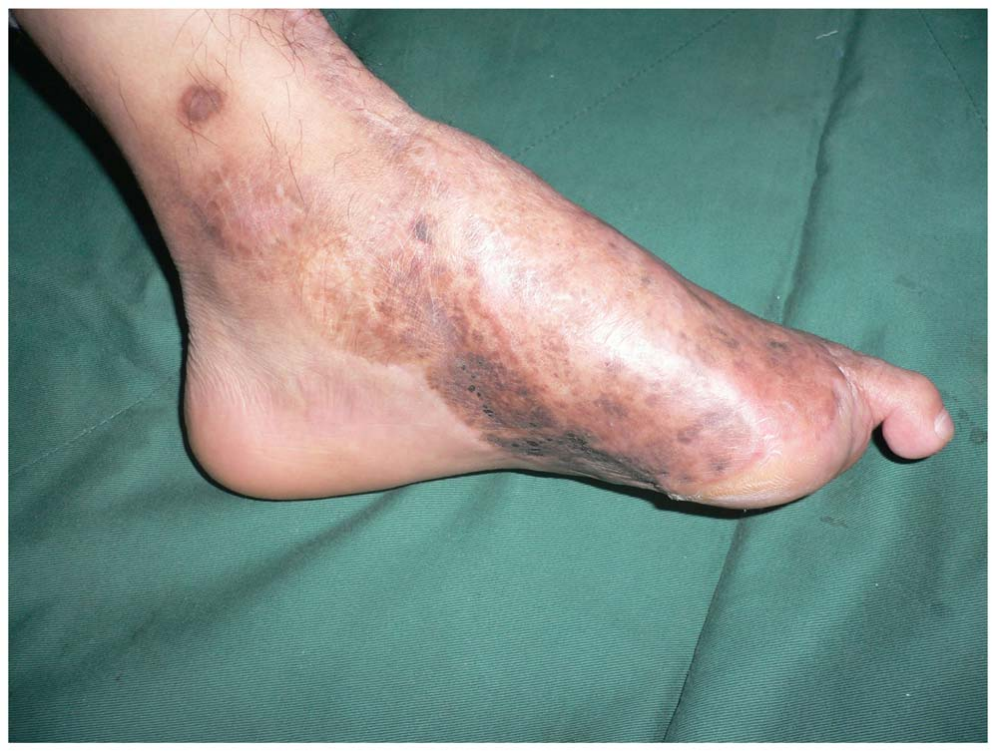
Appearance 14 months after debulking procedure.

A 32-year-old man (case number 2 in Table 1) suffered a soft tissue lesion of his left dorsal foot and weight-bearing sole with exposure of the transverse tarsal joint after a motor vehicle accident. He was admitted to our unit 10 days after the injury. Dislocation of the transverse tarsal joint had been treated elsewhere by open reduction and internal fixation with Kirschner wires. The size of the defect was 19×15 cm. It was successfully repaired with a 20×16 cm extended ALT septocutaneous perforator flap 6 days after admission. In this case, the microvascular anastomosis was performed end to end onto the tibialis anterior vessels and saphenous vein. The donor site was closed with a split-thickness skin grafting. The flap and donor sites healed without complication. Because the thick flap did not undergo a thinning procedure, the patient received a debulking procedure 3 months later. The whole flap was degloved to full thickness skin and then grafted in situ. Contour adaptation was good and the patient did not require custom shoes. No ulceration occurred during a 36 month follow-up (Fig. 1–5).

### Case 2

**Figure 6:**
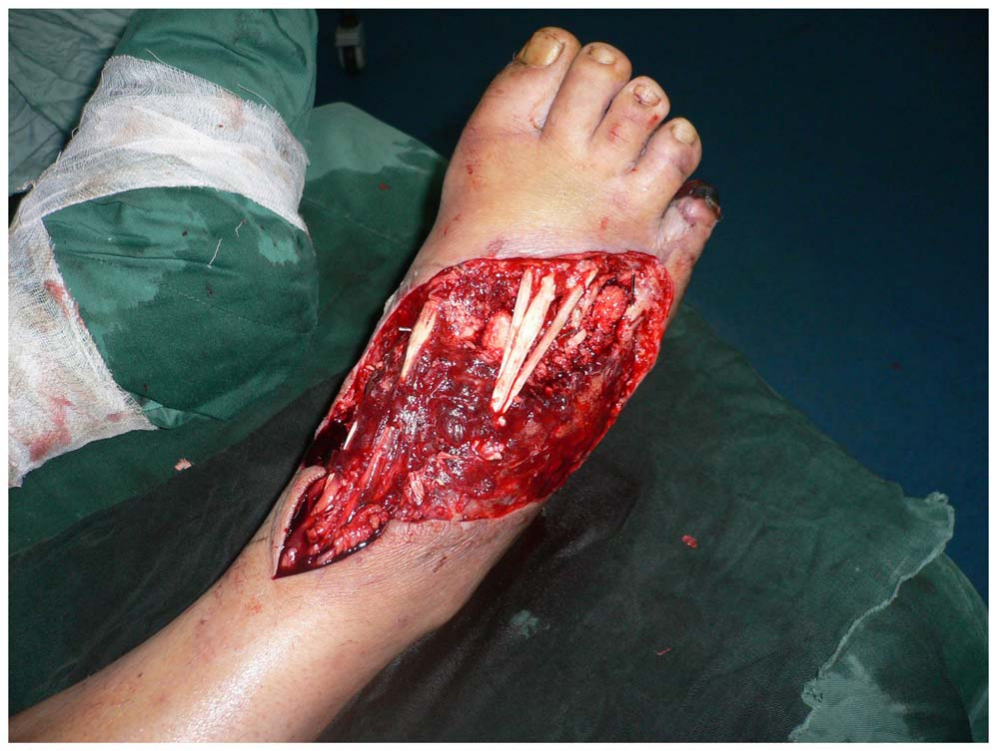
Preoperative view of extensive soft tissue defect of the dorsal foot with exposed tendon and bone.

**Figure 7:**
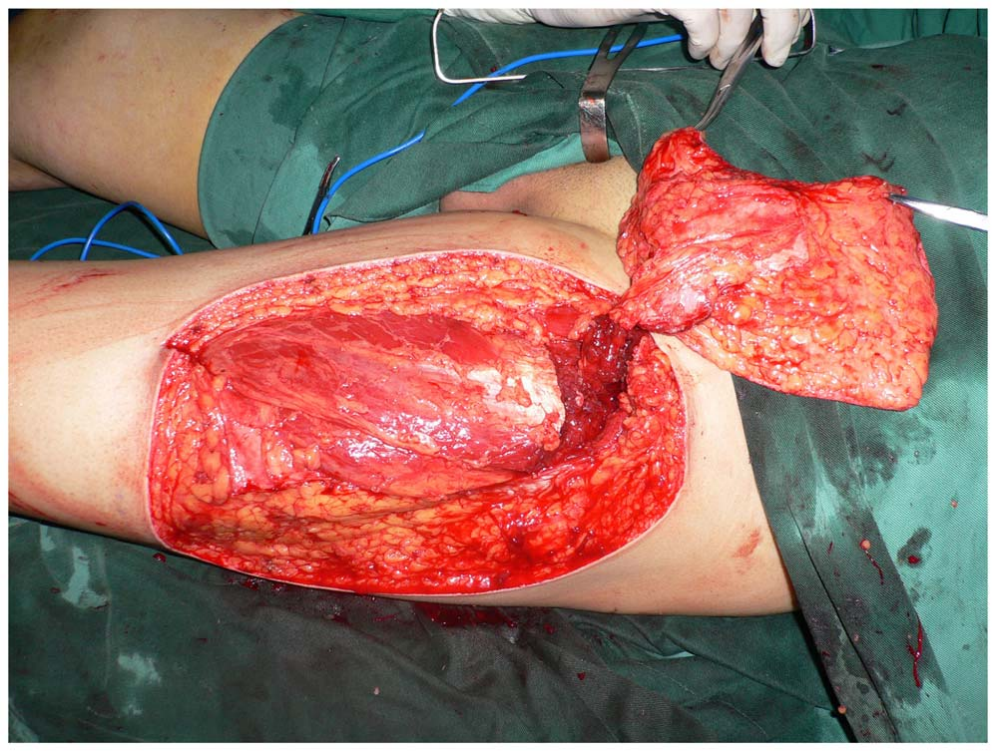
Intraoperative view after elevation of the thinned ALT musculocutaneous perforator flap.

**Figure 8:**
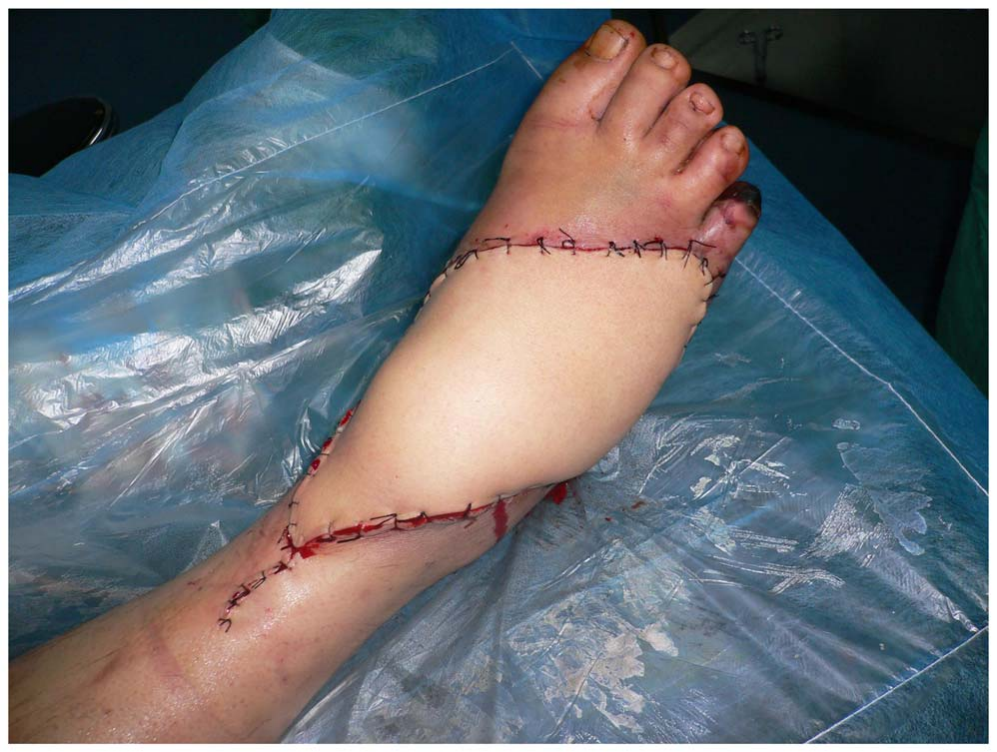
Early postoperative view of the flap.

A 27-year-old man (case number 8 in Table 1) had a 17×15 cm soft tissue defect of his right dorsal foot with exposure of the tendon and bone after a motor vehicle accident. This was repaired and covered with a 18×16 cm thinned extended ALT musculocutaneous perforator flap 10 days after the trauma. Split-thickness skin grafting was used for the donor site. Since only one perforator was located within the flap territory, a cuff of deep fascia was retained around the perforator. The vessel anastomosis was performed end to end onto the tibialis anterior vessels and saphenous vein. The flap survived completely and the result was satisfactory (Fig. 6–8).

### Case 3

**Figure 9:**
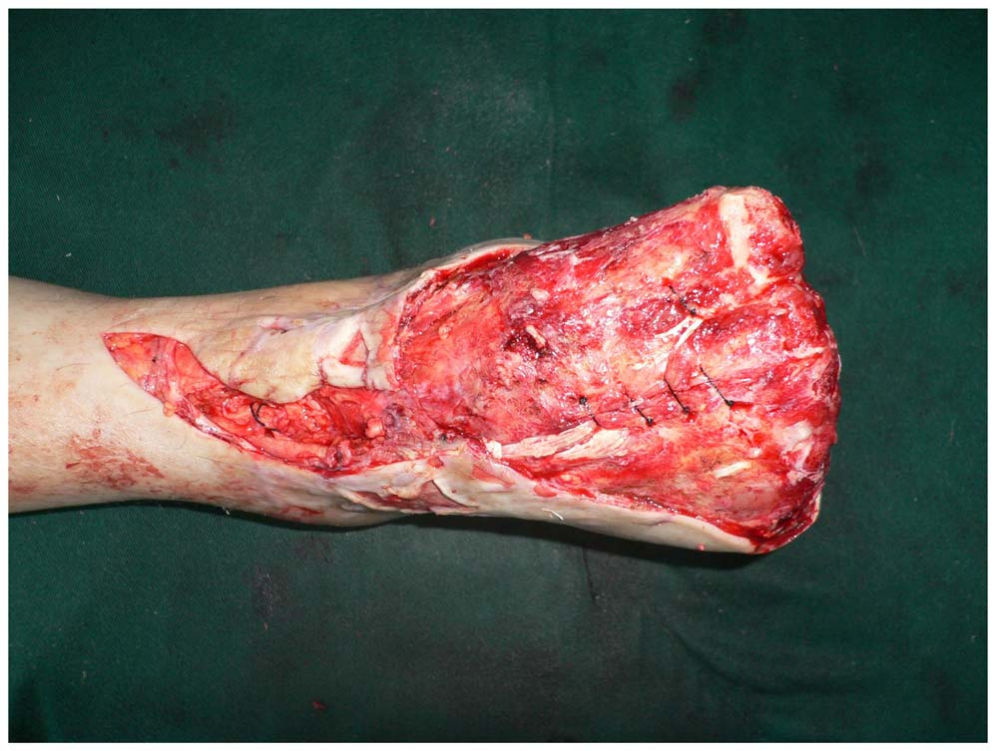
Preoperative view of extensive soft tissue defect of the degloved foot.

**Figure 10:**
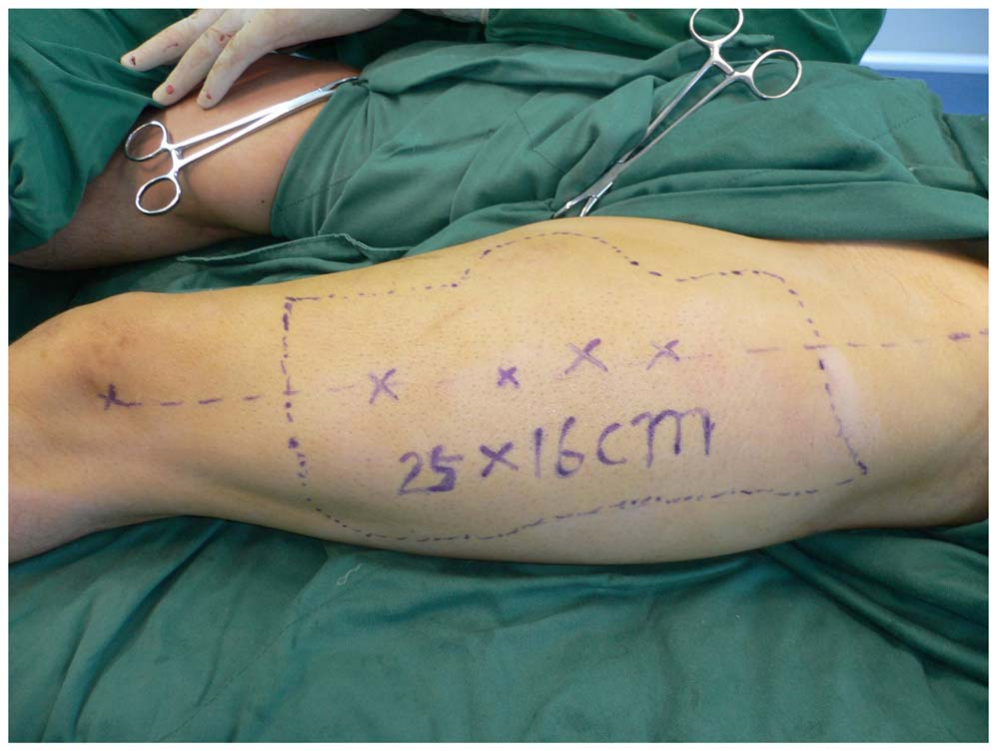
The thinned ALT flap (25×16 cm) was designed according to a template made from the area of the defect.

**Figure 11:**
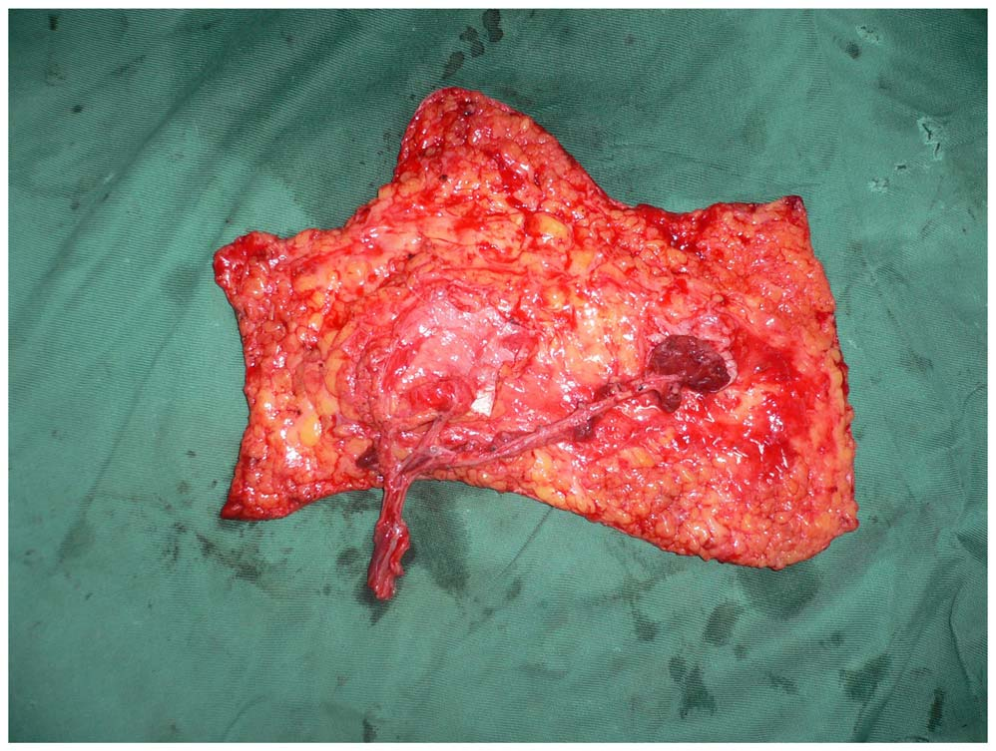
The elevated thinned ALT flap with three perforators surrounded by small cuffs of deep fascia.

**Figure 12:**
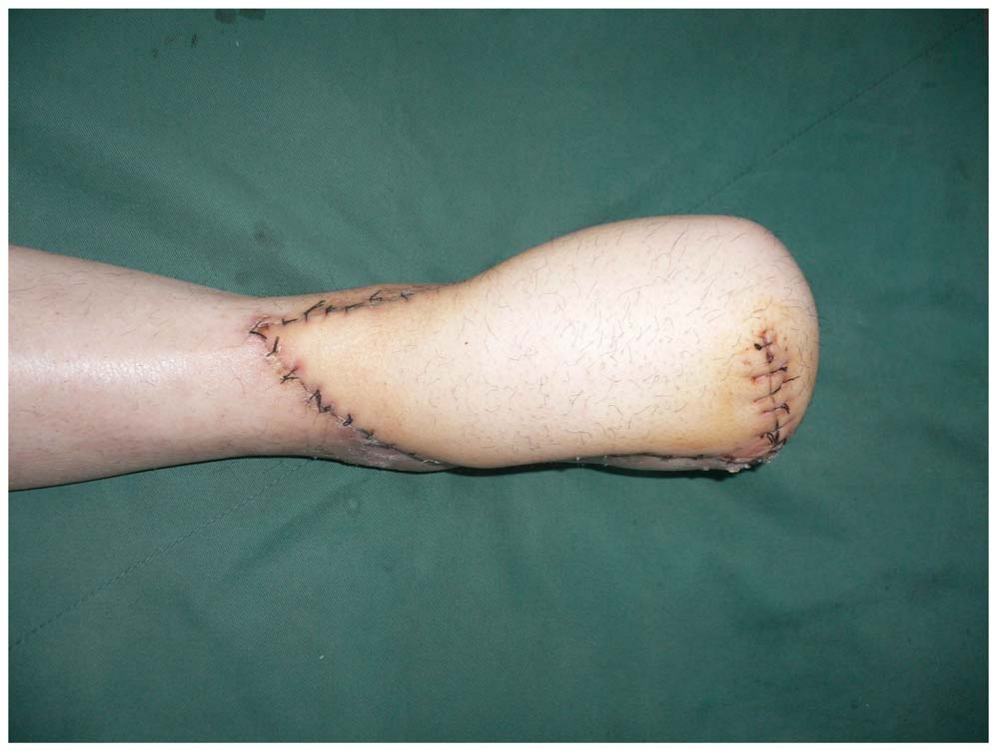
Appearance 12 days after operation with complete flap survival.

**Figure 13:**
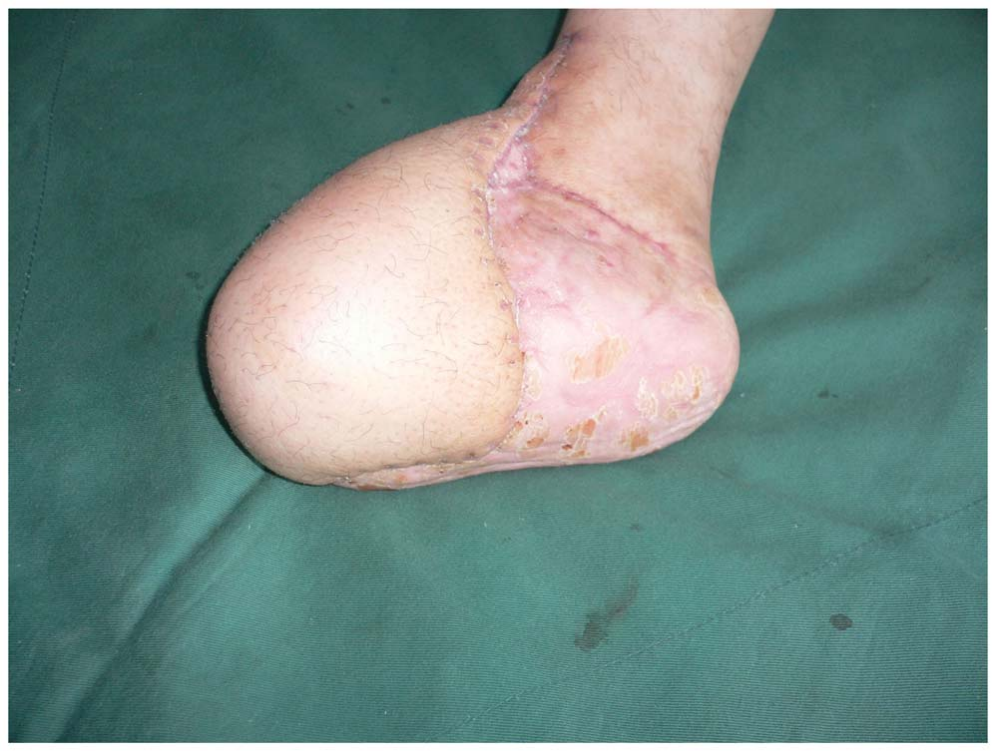
Three month follow-up with healed tissue envelope.

A 42-year-old man (case number 13 in Table 1) involved in a road traffic accident sustained a degloving injury to the right foot resulting in 24×15 cm defect with loss of all of the digits. This was repaired and covered with a 25×16 cm thinned extended ALT musculocutaneous perforator flap 9 days after the trauma. Three perforators were identified and dissected back to the descending lateral circumflex femoral vessel. A small cuff of deep fascia was kept around the cutaneous perforators to protect them. The vascular anastomosis was performed end to end onto the tibialis anterior vessels and saphenous vein, and split-thickness skin grafting was used for the donor site. The flap survived completely without any complications (Fig. 9–13).

### Case 4

**Figure 14:**
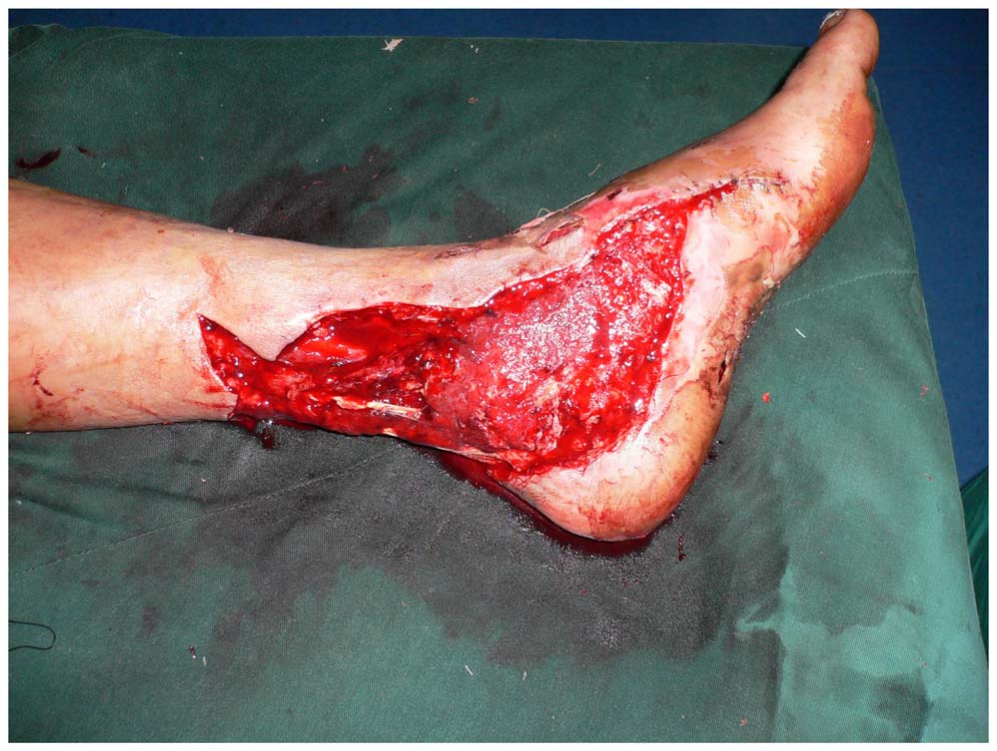
Preoperative view of extensive soft tissue defect over the hind foot and medial ankle.

**Figure 15:**
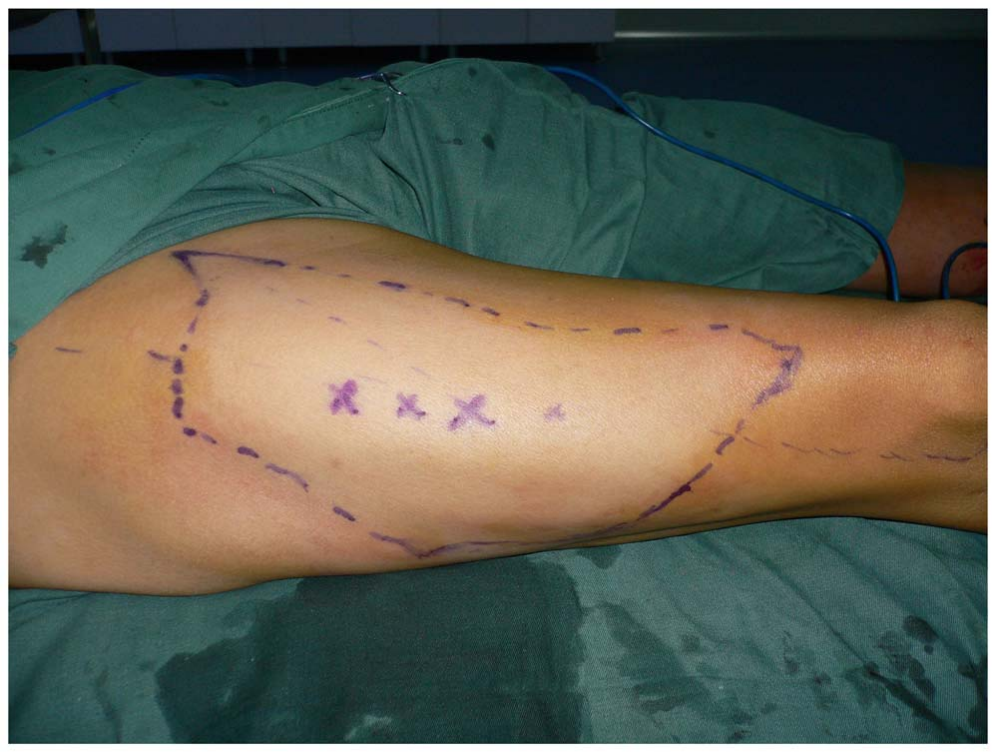
Design of the ALT flap (24×16 cm).

**Figure 16:**
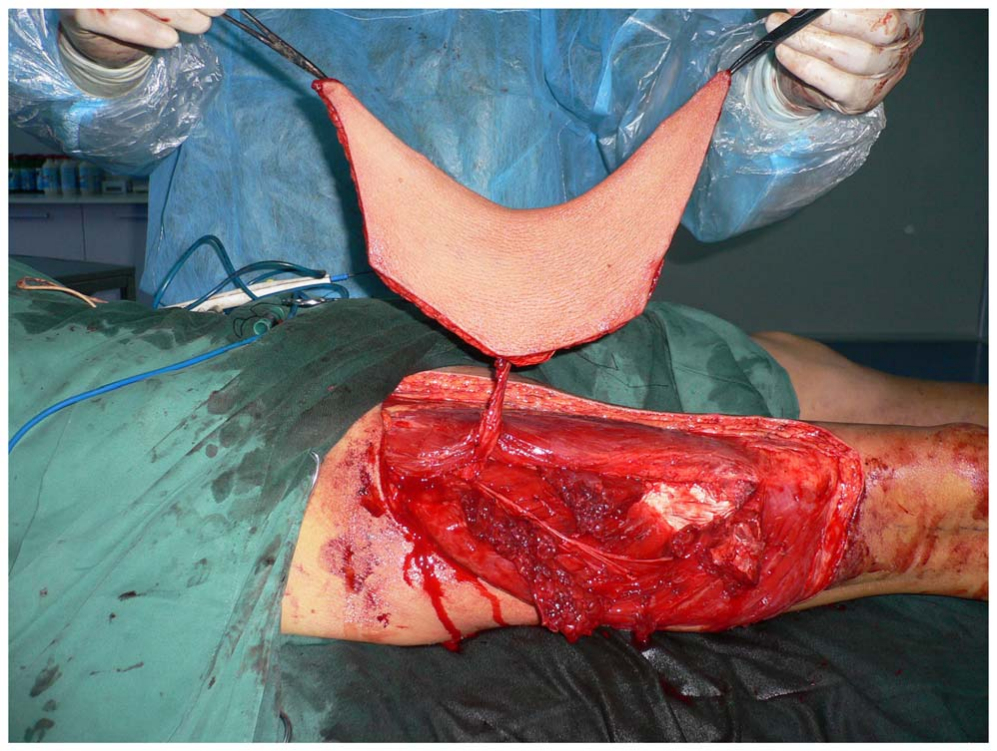
Intraoperative view after elevation of the ALT musculocutaneous perforator flap with the deep fascia.

**Figure 17:**
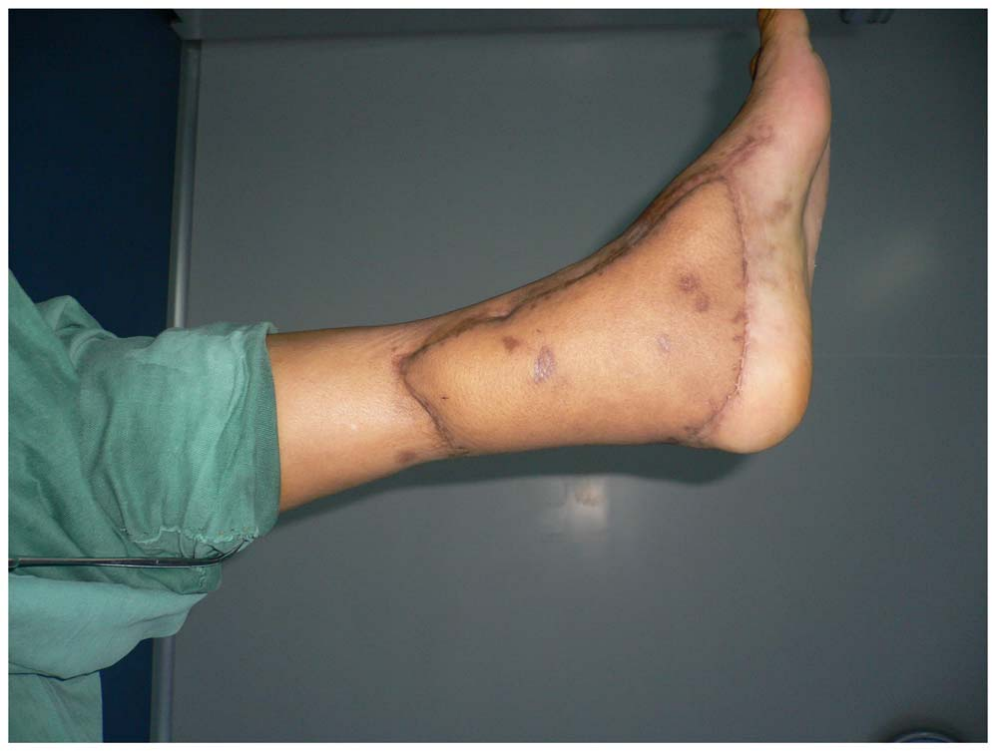
Two month follow-up with healed tissue envelope.

A 31-year-old man (case number 20 in Table 1) presented with a crush injury to the left foot, sustaining a soft tissue defect (23×15 cm) over the posterior foot and medial ankle. This was repaired and covered with a 24×16 cm extended ALT musculocutaneous perforator flap. Because of its analogous structure, the fascia of the flap was used as a substitute for a partial defect of the Achilles tendon 8 days after the trauma. The arterial anastomosis was performed end to end onto the tibialis posterior vessels and saphenous vein. Split-thickness skin grafting was used for the donor site. The flap and the donor site healed unevenly. The patient complained of fatigue while ascending and descending stairs. A visual gait analysis showed only minor abnormalities and the patient was not hindered from running (Fig. 14–17).

## Discussion

Fasciocutaneous or neurocutaneous flaps from the leg are useful and versatile reconstructive options in patients with soft tissue defects of the foot and ankle. These types of flaps are associated with less risk of failure, easier dissection and minimal risk for major vessel injury. However, some disadvantages of these flaps, like restricted rotation of short, wide adipofascial pedicles and limited dimensions, may limit their application for extensive defect reconstruction. Furthermore, these flaps are thick and often require a surgical revision. Therefore, they are best for when local tissues cannot provide adequate coverage and a free flap is indicated for optimum reconstruction.

Since the introduction of the ALT flap, it has become an important reconstructive method for patients with soft tissue defects of the foot and ankle. The ALT flap was first reported by Song et al [8] as a septocutaneous perforator flap in 1984 and has since gained popularity as a versatile flap in head and neck reconstruction, as used by Koshima et al [13]. After that, several authors have demonstrated that this type of flap is reliable and can be used for defects of the foot and ankle [4], [9], [10], [14].

The ALT flap is classically described as a perforator flap, which can be harvested to include skin only or skin and muscle, or as a chimeric flap with a separately perfused skin paddle. The main cutaneous perforators of the ALT flap derive from the descending branch of the lateral circumflex femoral artery. This descending branch runs downward either though the vastus lateralis muscle or into the intermuscular space between the rectus femoris and vastus lateralis muscle. It then terminates in the vastus lateralis muscle near the knee joint by branching into two to five cutaneous perforators at the lateral aspect of the thigh. Most of these perforators are found to exit within a 5-cm-diameter circle centered at the midpoint of the line between the anterior superior iliac crest and the superolateral border of the patella. The first perforator has the largest diameter [15]. Although there are many different classifications of perforators, they are usually classified as septocutaneous perforators or musculocutaneous perforators. Some authors have reported a small number of cases with absence of ALT flap perforators [16], [17]. In our series, all of the flaps had documented perforators. Although there are many methods to locate the perforators, in our experience a handheld Doppler probe has adequate sensitivity for perforator location.

The skin and subcutaneous adipose tissue of the foot and ankle is thin, and reconstruction of soft tissue in this region requires a thin flap to allow for a normal fit into footwear. One of the major advantages of the ALT flap is that it measures only 3 to 5 mm in thickness in slim patients. Even in overweight patients, it is possible for customized thinning to adapt to the defect of the foot and ankle. The rich blood supply of the suprafascial arterial plexus in the deep adipofascial tissue layer allows the fatty tissue in the superficial layer to be removed during ALT flap elevation [15]. The thinned ALT flap, first reported by Koshima et al [13], has been proven to be reliable in clinical application. The thinned flap also provides superior cosmetic and functional results in areas traditionally difficult to cover with thin, contoured free tissue, like the dorsum of the foot or over the Achilles tendon. When harvesting the thinned ALT flap, care must be taken to preserve a small cuff of deep fascia around large cutaneous perforators to protect them and their connection to the subdermal plexus, which supplies blood to the skin and superficial layer. Furthermore, there is a risk to venous drainage if the thinning is excessive and the polygonal venous network is injured [18]. Saint-Cyr et al believed that primary thinning should not be performed during extended ALT flap harvesting, in order to avoid flap failure [9]. In their series, they used a single perforator to support a relatively large fasciocutaneous vascular territory but the extended thinned flaps were designed to be perfused by two or three perforators in our series. If two separate perforators can not be located within the flap, a slightly large cuff of deep fascia should be retained around the perforator to secure the blood supply of the flap. Even then, partial flap necrosis occurred in one of our early patients who received excessive thinning. This demonstrated that only moderate thinning does not disrupt the linking vessels or reduce the vascular territory perfused within the extended ALT flap. The largest thinned flap in our series was 25×16 cm, and it survived completely without any complications. To the best of our knowledge, it is the largest thinned extended ALT flap ever reported. Patients with extensive defects of the foot and ankle often also experience loss of the Achilles tendon or extensor tendons. Another benefit of the ALT flap is that the fascia of the flap can be used as an ideal substitute for those tendons because of its analogous structure.

Since the inflammatory response of the traumatized lower extremity extends beyond the gross wound and results in perivascular changes in the blood vessels that can predispose the patient to thrombosis, anastomosis should be outside of the zone of injury during free flap transfer [4]. This requirement often influences the type of free flap that can be used. The vascular pedicle of the ALT flap is approximately 8 to 12 cm in length, which allows anastomosis to be performed “outside the zone of injury.” However, the long, narrow pedicle is vulnerable to compression throughout its course to the recipient vessels proximal to the ankle. Hemostasis and drainage should maintained adequately to prevent hematoma formation and the flap should be monitored carefully by clinical examination for color and capillary refill during the early postoperative period. In order to avoid flap failure, recipient vessel selection is also very important [19]. Although the vessels of the ALT flap match closely to the recipient vessels (dorsum pedis vessels, tibialis anterior vessels or tibialis posterior vessels) traumatic vascular impairment must be prevented by careful clinical examination intra-operatively. There are two veins of different sizes accompanying the descending branch of the lateral circumflex femoral artery. Many authors believed that only one accompanying vein anastomosis was enough for venous return [17], [20]. Since the flow strength of venous return sometimes differs between the two veins, unrelated to venous size, Kimata et al [17] prefers to check the quality of venous back-flow and choose an appropriate vein for anastomosis after the anastomosis of the artery has been completed. Rubino C [21] reported that flow rate measured postoperatively on flap arteries is significantly correlated with flap weight. In order to avoid congestion of the big flap and postoperative complications, we usually chose a superficial dorsum pedis vein or saphenous vein together with an accompanying vein of the artery as the two recipient veins for anastomosis. It secured the venous return of the flap and no flap congestion occurred in our series.

Eight anatomic types of the descending branch of the lateral circumflex femoral artery have been reported [17]. Technical Working with this versatility of the branch pattern of perforators when harvesting the ALT flap requires precise technical skills, including retrograde intramuscular isolation and dissection of small musculocutaneous perforators. We agree with the free-style flap harvest concept addressed by Wei and Mardini [22]. With this approach, any cutaneous perforators which can be located by a handheld Doppler probe can potentially be harvested by retrograde dissection as a free flap, regardless of regional anatomy. However, this has a higher potential risk for unintentional damage to the vessels and has a steep learning curve. All surgeries in our series were performed by experienced microsurgeons with expertise in this area.

There were no serious complications in our cases. Fatigue while ascending and descending stairs has been reported as a common donor site complication of ALT flaps in other studies [23], [24]. However, only one patient complained of this in our series. This can be attributed to meticulous dissection and minimal injury to the vastus lateralis muscle. Moreover, early physical therapy also plays an important role in minimizing weakness of the vastus lateralis. The major disadvantage of the flap is a less cosmetically acceptable donor site scar on the anterolateral thigh. In comparison with salvage reconstruction of the severely traumatized foot and ankle, this is considered negligible. Another limitation is the lack of research on sensory nerve coaptation of ALT flap. Since in our series, most of the defects in the foot and ankle are of non weight-bearing area and no foot ulceration occurred after coverage of the defects. Moreover, Santanelli [25] reported that, flaps without surgical nerve repair showed progressive improvement of sensitive thresholds, achieving a good protective sensibility, similar to the flaps with nerve reconstruction, only lacking two-point discrimination or dermatomic somatosensory-evoked potentials. Although all the flaps in our series have no nerve repair, the harmful effect on the free flap reconstruction of the sole of the foot might be minor after 12 months. The sensate ALT flap is typically described as innervated by the lateral cutaneous femoral nerve. Two other nerves, the superior perforator nerve and the median perforator nerve, which enter the flap at its medial border, might have a role in ALT flap innervations [26]. Readers can make further research on the flaps with sensory nerve coaptation and an increase functional recovery might be expected.

## Conclusion

The extended ALT flap, despite its large dimensions, has been useful and safe in our experience. It provides another good alternative for reconstruction of the foot and ankle, in addition to fasciocutaneous or neurocutaneous flaps. This study also supports the high reliability and excellent vascular supply of moderate thinned extended ALT flaps.
